# *Candida albicans* renal abscesses predisposed by staghorn calculi

**DOI:** 10.1590/0037-8682-0421-2023

**Published:** 2023-10-13

**Authors:** Chee Yik Chang

**Affiliations:** 1 Infectious Disease Unit, Medical Department, Hospital Sultanah Aminah, Johor, Malaysia.

**FIGURE 1:**
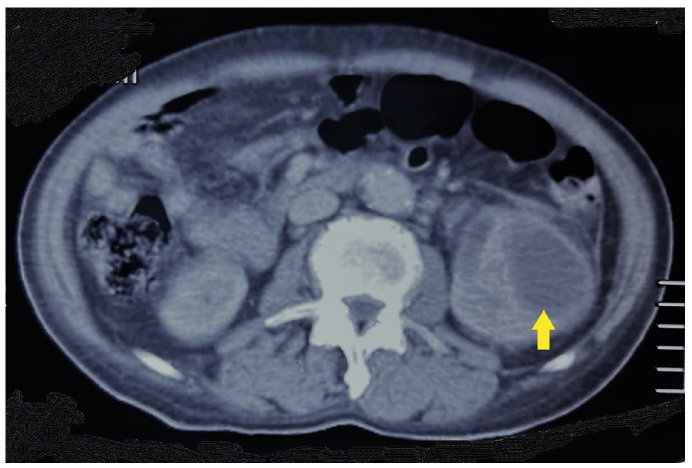
Computed tomography of the abdomen and pelvis showing well-defined collections in the left kidney resembling abscesses, with the largest collection measuring 3.7 cm × 3.8 cm × 3.9 cm at the lower pole (indicated by arrow).

A 73-year-old man with type 2 diabetes mellitus and hypertension presented with fever, lethargy, and reduced oral intake for two weeks. The patient was febrile and hemodynamically stable upon arrival. Otherwise, physical examination results were unremarkable. Blood cultures revealed no growth, whereas urine cultures revealed *Candida albicans*. Abdominal and pelvic computed tomography (CT) revealed left renal abscesses, left staghorn calculi, and hydroureter (Figure 1). The left renal abscess was aspirated and the pus was drained, revealing *Candida albicans*, which is sensitive to fluconazole. The patient underwent left retrograde pyelography and ureteric stenting. The patient was initially treated with intravenous fluconazole, followed by oral fluconazole for four weeks. Follow-up CT revealed resolution of the renal abscess.

Renal abscesses caused by *Candida albicans*, an opportunistic fungal pathogen, are rare but serious manifestations of invasive candidiasis. These abscesses typically occur in immunocompromised individuals such as those with diabetes mellitus, long-term antibiotic use, or urinary tract abnormalities^1^. Candida-related renal abscesses are characterized by localized pus pockets within the kidney parenchyma and are frequently accompanied by fever, flank pain, and sepsis^1^ ^,^ ^2^. Early detection using imaging techniques such as CT and prompt antifungal therapy (usually fluconazole) are critical to avoid potential complications such as renal failure or systemic dissemination^3^.
